# Aortic arch surgery for DeBakey type 1 aortic dissection in patients aged 60 years or younger

**DOI:** 10.1093/bjsopen/zrae047

**Published:** 2024-05-20

**Authors:** Fausto Biancari, Javier Rodriguez Lega, Giovanni Mariscalco, Sven Peterss, Joscha Buech, Antonio Fiore, Andrea Perrotti, Andreas Rukosujew, Angel G Pinto, Till Demal, Konrad Wisniewski, Marek Pol, Giuseppe Gatti, Igor Vendramin, Mauro Rinaldi, Robert Pruna-Guillen, Dario Di Perna, Zein El-Dean, Hiwa Sherzad, Francesco Nappi, Mark Field, Matteo Pettinari, Mikko Jormalainen, Angelo M Dell’Aquila, Francesco Onorati, Eduard Quintana, Tatu Juvonen, Timo Mäkikallio

**Affiliations:** Department of Medicine, South-Karelia Central Hospital, University of Helsinki, Lappeenranta, Finland; Heart and Lung Center, Helsinki University Hospital, University of Helsinki, Helsinki, Finland; Cardiovascular Surgery Department, University Hospital Gregorio Marañón, Madrid, Spain; Department of Cardiac Surgery, Glenfield Hospital, Leicester, UK; Department of Cardiac Surgery, LMU University Hospital, Ludwig Maximilian University, Munich, Germany; Department of Cardiac Surgery, LMU University Hospital, Ludwig Maximilian University, Munich, Germany; German Centre for Cardiovascular Research, Partner Site Munich Heart Alliance, Munich, Germany; Department of Cardiac Surgery, Hôpitaux Universitaires Henri Mondor, Assistance Publique-Hôpitaux de Paris, Creteil, France; Department of Thoracic and Cardiovascular Surgery, University of Franche-Comte, Besancon, France; Department of Cardiothoracic Surgery, University Hospital Muenster, Muenster, Germany; Cardiovascular Surgery Department, University Hospital Gregorio Marañón, Madrid, Spain; Department of Cardiovascular Surgery, University Heart and Vascular Center Hamburg, Hamburg, Germany; Department of Cardiothoracic Surgery, University Hospital Muenster, Muenster, Germany; Department of Cardiac Surgery, Third Faculty of Medicine, Charles University and University Hospital Kralovske Vinohrady, Prague, Czech Republic; Division of Cardiac Surgery, Cardio-thoracic and Vascular Department, Azienda Sanitaria Universitaria Giuliano Isontina, Trieste, Italy; Cardiothoracic Department, University Hospital, Udine, Italy; Cardiac Surgery, Molinette Hospital, University of Turin, Turin, Italy; Department of Cardiovascular Surgery, Hospital Clínic de Barcelona, University of Barcelona, Barcelona, Spain; Department of Cardiac Surgery, Centre Hospitalier Annecy Genevois, Epagny Metz-Tessy, France; Department of Cardiac Surgery, Glenfield Hospital, Leicester, UK; Department of Cardiac Surgery, Glenfield Hospital, Leicester, UK; Department of Cardiac Surgery, Centre Cardiologique du Nord de Saint-Denis, Paris, France; Liverpool Centre for Cardiovascular Sciences, Liverpool Heart and Chest Hospital, Liverpool, UK; Department of Cardiac Surgery, Ziekenhuis Oost Limburg, Genk, Belgium; Heart and Lung Center, Helsinki University Hospital, University of Helsinki, Helsinki, Finland; Department of Cardiothoracic Surgery, University Hospital Muenster, Muenster, Germany; Department of Cardiac Surgery, Martin Luther University Halle-Wittenberg, Halle, Germany; Division of Cardiac Surgery, University of Verona Medical School, Verona, Italy; Department of Cardiovascular Surgery, Hospital Clínic de Barcelona, University of Barcelona, Barcelona, Spain; Heart and Lung Center, Helsinki University Hospital, University of Helsinki, Helsinki, Finland; Research Unit of Surgery, Anesthesia and Critical Care, University of Oulu, Oulu, Finland; Department of Medicine, South-Karelia Central Hospital, University of Helsinki, Lappeenranta, Finland

## Abstract

**Background:**

Extended aortic repair is considered a key issue for the long-term durability of surgery for DeBakey type 1 aortic dissection. The risk of aortic degeneration may be higher in young patients due to their long life expectancy. The early outcome and durability of aortic surgery in these patients were investigated in the present study.

**Methods:**

The subjects of the present analysis were patients under 60 years old who underwent surgical repair for acute DeBakey type 1 aortic dissection at 18 cardiac surgery centres across Europe between 2005 and 2021. Patients underwent ascending aortic repair or total aortic arch repair using the conventional technique or the frozen elephant trunk technique. The primary outcome was 5-year cumulative incidence of reoperation on the distal aorta.

**Results:**

Overall, 915 patients underwent surgical ascending aortic repair and 284 patients underwent surgical total aortic arch repair. The frozen elephant trunk procedure was performed in 128 patients. Among 245 propensity score–matched pairs, total aortic arch repair did not decrease the rate of distal aortic reoperation compared to ascending aortic repair (5-year cumulative incidence, 6.7% *versus* 6.7%, subdistributional hazard ratio 1.127, 95% c.i. 0.523 to 2.427). Total aortic arch repair increased the incidence of postoperative stroke/global brain ischaemia (25.7% *versus* 18.4%, *P* = 0.050) and dialysis (19.6% *versus* 12.7%, *P* = 0.003). Five-year mortality was comparable after ascending aortic repair and total aortic arch repair (22.8% *versus* 27.3%, *P* = 0.172).

**Conclusions:**

In patients under 60 years old with DeBakey type 1 aortic dissection, total aortic arch replacement compared with ascending aortic repair did not reduce the incidence of distal aortic operations at 5 years. When feasible, ascending aortic repair for DeBakey type 1 aortic dissection is associated with satisfactory early and mid-term outcomes.

**Trial registration:**

ClinicalTrials.gov Identifier: NCT04831073.

## Teaser

In patients under 60 years old with acute DeBakey type 1 aortic dissection, when feasible, ascending aortic repair compared to total aortic arch repair is associated with favourable early and mid-term outcomes.

## Introduction

Surgical and perfusion strategies for acute type A aortic dissection (TAAD) are topics of intense clinical research aiming to identify strategies to improve early postoperative outcomes and to guarantee the long-term durability of the operation^1^. TAAD involving the aortic arch, that is DeBakey type 1 aortic dissection, poses the dilemma of whether to perform a complete resection of the aortic arch with or without repair of the proximal part of the descending thoracic aorta with hybrid prostheses. Such an extensive surgical approach, often associated with completion endovascular treatment^2^, is thought to reduce the risk of distal progression of the disease by favouring the remodelling of the dissected downstream aorta and thus preventing aortic wall degeneration. This extensive surgical approach is attractive in young patients to reduce the incidence of distal aortic complications during their long lifespan^3^. However, data on the efficacy of total aortic arch repair in young patients are scarce^3–7^. In this multicentre study, whether total aortic arch repair may reduce the need for distal aortic reoperation in patients <60 years old with DeBakey type 1 aortic dissection was evaluated.

## Patients and methods

### Study population

The European Registry of Type A Aortic Dissection (ERTAAD)^8^ is a retrospective, multicentre study including consecutive patients who underwent surgical repair of the thoracic aorta for acute TAAD at 18 cardiac surgery centres in eight European countries (Belgium, Czech Republic, Finland, France, Germany, Italy, Spain and the UK) from January 2005 to March 2021. Eleven hospitals provided data on consecutive patients operated later than 2005 because of lack of availability of patients’ records in the early study period.

The Ethical Review Board of the Helsinki University Hospital, Finland (21 April 2021, diary no. HUS/237/2021) and the Ethical Review Board of each participating hospital approved this study. The requirement for informed consent was waived because of the retrospective nature of this study.

The inclusion criteria of the ERTAAD registry were the following: patients with acute TAAD; patients over 18 years old; onset of symptoms within 7 days prior to surgery; primary surgical repair of acute TAAD; and any other major cardiac surgical procedure concomitant with surgery for TAAD^8^. The exclusion criteria were the following: patients aged under 18 years; onset of symptoms more than 7 days prior to surgery; prior procedure for TAAD; retrograde TAAD; concomitant endocarditis; and TAAD secondary to blunt or penetrating chest trauma^8^. For the purpose of this study, only patients under 60 years old and with dissection involving the aortic arch, that is DeBakey type 1 aortic dissection, were included. As the aim of this study was to evaluate the impact of total aortic arch repair compared to aortic repair limited to the ascending aorta, patients with partial repair of the aortic arch were excluded from this study.

The rationale for this subanalysis is based on the analysis of 3293 patients with DeBakey type 1 aortic dissection, which showed that patients aged under 60 years old, when adjusted for total aortic arch repair, had a significantly higher cumulative incidence of distal aortic reoperation (10 year, 10.3% *versus* 7.0%, subdistributional hazard ratio (SHR) 1.458, 95% c.i. 1.097 to 1.937) and significantly lower mortality rate (10-year, 31.4% *versus* 59.9%, HR 0.484, 95% c.i. 0.426 to 0.551) compared to older patients. Furthermore, the previous analyses showed that the 10-year expected survival of a country-, year-, age- and sex-matched general population with 3-month TAAD survivors was 97.5% in subjects aged under 50 years and 92.6% for those aged 50–59 years, while these measures decreased markedly in patients aged over 60 years^8^. Because the 10-year life-expectancy of patients aged under 50 years and those aged 50–59 were both similarly high, the current analysis included patients aged less than 60 years.

Surgical procedures were classified as follows. Ascending aortic repair: surgical repair of the ascending aorta without resection of a part of the aortic arch requiring revascularization of any of the epiaortic vessels. The hemiarch repair, that is bevelled anastomosis with resection of the small curvature of the aortic arch, was considered as an ascending aortic repair. Conventional total aortic arch repair: surgical repair of the entire aortic arch with conventional vascular prosthesis associated with revascularization of the epiaortic vessels and distal aortic anastomosis to the Ishimaru zones 3–4. The frozen elephant trunk procedure: surgical repair of the aortic arch and the descending thoracic aorta using commercially available hybrid prosthesis. Aortic root replacement: replacement of the aortic root according to the Bentall–DeBono technique or aortic valve sparing aortic root replacement according to David’s or Yacoub’s techniques.

### Study outcomes

The primary outcome of this study was 5-year cumulative incidence of reoperation on the distal aorta, which refers to surgical and/or endovascular reintervention on the downstream aorta beyond the ascending aorta. Additional endovascular procedures performed during the primary procedure or during the index hospitalization were not considered repeat distal aortic procedures. Distal aortic reinterventions included surgical replacement or stenting/stent-grafting of one or more aortic segments distal to the ascending aorta as well as embolization of aortic pseudoaneurysm and stent-grafting of aortic branches. Embolization of aortic pseudoaneurysm and stent-grafting of aortic branches were considered distal reinterventions of interest because primary extensive surgical or hybrid repair might favour aortic remodelling and prevent the development of these dissection-related complications. Additional data were also provided on distal aortic reoperations at 10-year follow-up.

Secondary outcomes were in-hospital mortality during index hospitalization, stroke/global brain ischaemia, paraplegia/paraparesis, mesenteric ischaemia, sepsis, dialysis, heart failure and reoperation for intrathoracic bleeding. A composite outcome including in-hospital mortality, stroke and/or global brain ischaemia as well as 5-year mortality and proximal aortic procedures involving the ascending aorta, aortic root and/or aortic valve were other secondary outcomes of this analysis. The definition criteria of these outcomes have been reported previously^8^. Data on the date of death and repeated aortic intervention were collected retrospectively from electronic institutional and national registries as well as by contacting regional hospitals, patients and their relatives.

### Statistical analysis

Continuous variables were reported as means and standard deviations. Categorical variables were reported as counts and percentages. The chi-square and the Fisher’s exact tests were used to analyse differences of categorical variables, and the Mann–Whitney test and the Kruskal–Wallis test were used to compare continuous variables between the study groups. Because complications requiring aortic reoperations might be hindered by a patient’s death occurring during the study period, competing risk analyses using the Fine–Gray test with all-cause death as a competing event were performed to estimate the SHRs and 95% confidence intervals for cumulative incidence of distal and proximal aortic reoperations. Propensity score-matching analysis was performed to adjust for imbalances in baseline characteristics between the ascending aortic repair and the total aortic arch repair study groups. A multilevel mixed-effects logistic regression was performed to estimate a propensity score considering any cluster effect related to the participating hospitals, with the type of procedure as a dependent variable and including the following baseline and operative variables as covariates: age, sex, genetic aortic syndrome, bicuspid aortic valve, iatrogenic TAAD, diabetes, stroke, pulmonary disease, extracardiac arteriopathy, prior cardiac surgery, preoperative cardiac massage, cardiogenic shock requiring inotropes, invasive mechanical ventilation, cerebral malperfusion, spinal malperfusion, renal malperfusion, mesenteric malperfusion, peripheral malperfusion, salvage procedure, tear located in the aortic arch, aortic root replacement procedure, coronary surgery and mitral or tricuspid valve surgery. Propensity score matching was performed using the *psmatch2* module for Stata with a caliper width of 0.2 the standard deviation of the logit, that is 0.22. Standardized difference under 0.1 was considered a non-significant imbalance of the covariates between the study groups. The outcomes in the ascending aortic repair, conventional total aortic arch repair and the frozen elephant trunk repair study groups were adjusted by multilevel mixed-effects logistic regression including the above-listed covariates and considering the cluster effect of participating hospitals. Survival analysis was performed using the Kaplan–Meier method and the multivariable multilevel mixed-effects parametric survival model. Statistical analyses were performed with SPSS (version 29.0, SPSS Inc., IBM, Chicago, Illinois, USA) and Stata (version 15.1, StataCorp LLC, College Station, Texas, USA) statistical software.

## Results

### Study population

**Fig. 1:**
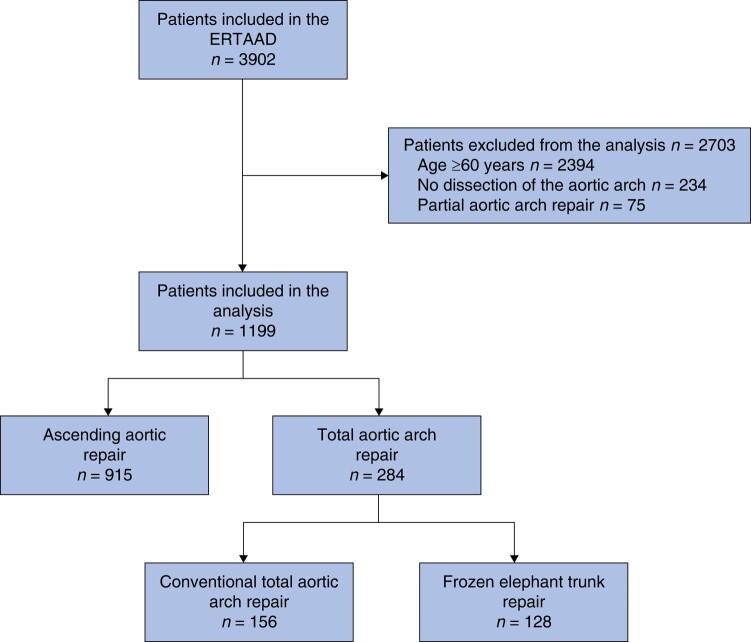
Study flowchart.

ERTAAD, European Registry of Type A Aortic Dissection; SHR, subdistributional hazard ratio.

A total of 3902 consecutive patients were included in the ERTAAD. Among 1508 patients under 60 years old, dissection involved the aortic arch in 1274 (84.5%). Seventy-five patients underwent partial aortic arch repair and were excluded from this analysis. Overall, 1199 patients with DeBakey type 1 aortic dissection fulfilled the inclusion criteria of this study and were the subjects of the present analysis (*Fig. 1*). Baseline characteristics of the patients are shown in *Table 1*.

**Table 1:** Patients’ characteristics and operative data of patients in the study groups

	Unmatched patients			Matched patients		
Variables	Ascending aortic repair	Total aortic arch repair	Standardized differences	Ascending aortic repair	Total aortic arch repair	Standardized differences
	(*n* = 915)	(*n* = 284)		(*n* = 245)	(*n* = 245)	
**Baseline characteristics**						
Age (years), mean(s.d.)	50.5(7.6)	49.4(8.4)	0.134	50.0(7.7)	49.6(8.5)	0.042
Female	176 (19.2)	43 (15.1)	0.109	35 (14.3)	38 (15.5)	0.034
eGFR, mean(s.d.), ml/min 1.73 m^2^	75(24)	79(25)	0.156	76(25)	79(24)	0.108
Genetic aortic syndrome	39 (4.3)	16 (5.6)	0.063	16 (6.5)	14 (5.7)	0.034
Bicuspid aortic valve	64 (7.0)	13 (4.6)	0.104	12 (4.9)	13 (5.3)	0.019
Iatrogenic dissection	14 (1.5)	0 (0)	0.176	3 (1.2)	0 (0)	0.157
Diabetes	17 (1.9)	7 (2.5)	0.042	4 (1.6)	5 (2.0)	0.030
Stroke	31 (3.4)	6 (2.1)	0.078	6 (2.4)	6 (2.4)	0.000
Pulmonary disease	49 (5.4)	9 (3.2)	0.108	11 (4.5)	9 (3.7)	0.041
Extracardiac arteriopathy	27 (3.0)	8 (2.8)	0.008	6 (2.4)	8 (3.3)	0.049
Prior cardiac surgery	12 (1.3)	5 (1.8)	0.037	3 (1.2)	0 (0)	0.157
Cardiac massage	35 (3.8)	10 (3.5)	0.016	8 (3.3)	8 (3.3)	0.000
Shock requiring inotropes	123 (13.4)	43 (15.1)	0.049	31 (12.7)	35 (14.3)	0.048
Invasive mechanical ventilation	73 (8.0)	20 (7.0)	0.036	14 (5.7)	17 (6.9)	0.050
Preoperative malperfusion						
Cerebral	197 (21.5)	67 (23.6)	0.049	53 (21.6)	59 (24.1)	0.058
Spinal	23 (2.5)	13 (4.6)	0.112	10 (4.1)	8 (3.3)	0.043
Renal	87 (9.5)	33 (11.6)	0.069	14 (5.7)	13 (5.3)	0.038
Mesenteric	40 (4.4)	33 (11.6)	0.073	14 (5.7)	13 (5.3)	0.018
Peripheral	175 (19.1)	50 (17.6)	0.039	33 (13.5)	45 (18.4)	0.134
**Operative data**						
Salvage procedure	36 (3.9)	10 (3.5)	0.022	7 (2.9)	8 (3.3)	0.024
Tear in the aortic arch	101 (11.0)	115 (40.5)	0.715	74 (30.2)	76 (31.0)	0.018
Aortic root replacement	357 (39.0)	92 (32.4)	0.139	85 (34.7)	88 (35.9)	0.026
Coronary surgery	89 (9.7)	18 (6.3)	0.125	13 (5.3)	18 (7.3)	0.084
Mitral or tricuspid valve surgery	3 (0.3)	0 (0)	0.081	2 (0.8)	0 (0)	0.128
Aortic cross-clamp time, mean(s.d.), min	119(56)	164(73)	0.690	116(62)	167(75)	0.733
Cardiopulmonary bypass time, mean(s.d.), min	213(83)	272(101)	0.636	209(78)	276(101)	0.742
TEVAR during primary procedure or the index hospitalization	5 (0.5)	9 (3.2)	0.195	1 (0.4)	8 (3.3)	0.214

Continuous values are reported as mean and standard deviation (in parentheses). Categorical variables are reported as counts and percentages (in parentheses). eGFR, estimated glomerular filtration rate according to the CKD-EPI equation; TEVAR, thoracic endovascular aortic repair.

Time from onset of symptoms to surgery was a mean of 16(15) h (median 7 h; data available in 917 patients). Total aortic arch repair was performed in 284 patients, with a conventional surgical prosthesis in 156 patients and with the frozen elephant trunk technique in 128 patients. The Thoraflex hybrid prosthesis (Terumo Corporation, Tokyo, Japan) was used in 120 patients, the E-Vita hybrid prosthesis (Artivion, Kennesaw, GA, USA) in seven patients, and information on the hybrid prosthesis used was not available in one patient.

The mean(s.d.) follow-up of these patients was 4.7(4.5) years. During a 5-year postoperative period, 65 patients required a total of 86 distal aortic endovascular and/or surgical operations, which are summarized in *Table 2*. Sixty-seven distal aortic reoperations were performed in 49 patients whose primary procedure was ascending aortic repair, 16 distal aortic reoperations were performed in 13 patients who had undergone conventional total aortic arch repair and three distal aortic reoperations were performed in three patients who had undergone the frozen elephant trunk procedure (*Table 2*).

**Table 2:** Distal aortic procedures in the study groups during a 5-year follow-up period

Procedures	Ascending aortic repair	Conventional total aortic arch repair	Frozen elephant trunk procedure	Total
	(49/915 patients)	(13/156 patients)	(3/128 patients)	
TEVAR	19	6	2	27
Frozen elephant trunk procedure	14	2	0	16
Conventional total aortic arch repair	9	3	0	12
EVAR	9	3	0	12
Partial aortic arch repair	4	0	1	5
Open repair of the descending thoracic aorta	4	0	0	4
Surgical local repair	1	2	0	3
Open repair of the thoracoabdominal aorta	2	0	0	2
Open repair of the abdominal aorta	2	0	0	2
Renal artery stenting	1	0	0	1
Stent-grafting of the axillary artery	1	0	0	1
Embolization of aortic pseudoaneurysm	1	0	0	1
Total	67	16	3	86

EVAR, endovascular repair of the abdominal aorta; TEVAR, thoracic endovascular aortic repair.

The outcomes of unmatched patients who underwent ascending aortic repair and those who underwent total aortic arch repair are summarized in *Table 3*. Total aortic arch repair did not reduce the incidence of distal aortic reoperations (at 5 years, cumulative incidence 6.9% *versus* 6.8%; unadjusted SHR 1.063, 95% c.i. 0.605 to 1.869; at 10 years, cumulative incidence 11.0% *versus* 10.1%; unadjusted SHR 1.026, 95% c.i. 0.629 to 1.675). Total aortic arch repair was associated with an increased rate of postoperative dialysis, whereas there were no differences in other early and late outcomes (*Table 3*).

**Table 3:** Early and late outcomes of patients in the study groups

	Unmatched patients			Matched patients		
	Ascending aortic repair	Total aortic arch repair	*P*	Ascending aortic repair	Total aortic arch repair	*P*
	(*n* = 915)	(*n* = 284)		(*n* = 245)	(*n* = 245)	
**Early outcomes**						
In-hospital death	109 (11.9)	42 (14.8)	0.202	29 (11.8)	40 (16.3)	0.153
Stroke/global brain ischaemia	177 (19.3)	68 (23.9)	0.093	45 (18.4)	63 (25.7)	0.050
Composite outcome	238 (26.0)	89 (31.3)	0.078	62 (25.3)	83 (33.9)	0.038
Paraparesis/paraplegia	51 (5.6)	23 (8.1)	0.122	15 (6.1)	21 (8.6)	0.299
Mesenteric ischaemia	38 (4.2)	11 (3.9)	0.833	7 (2.9)	9 (3.7)	0.611
Sepsis	112 (12.2)	42 (14.8)	0.262	29 (11.8)	36 (14.7)	0.351
Dialysis	119 (13.0)	57 (20.1)	0.003	31 (12.7)	48 (19.6)	0.003
Reoperation for bleeding	118 (12.9)	42 (14.8)	0.413	30 (12.2)	36 (14.7)	0.427
Heart failure	110 (12.0)	36 (12.7)	0.768	26 (10.6)	34 (13.9)	0.270
**5-year outcomes**						
Distal aortic reoperation	49 (6.8)	16 (6.9)	0.832	12 (6.7)	14 (6.7)	0.759
Proximal aortic reoperation	34 (4.8)	6 (2.3)	0.202	8 (4.2)	5 (2.3)	0.355
Mortality	168 (21.2)	65 (25.9)	0.082	46 (22.8)	59 (27.3)	0.172

Values are *n* (%). Composite outcome: in-hospital death, stroke and/or global brain ischaemia.

### Ascending aortic repair *versus* total aortic arch repair—propensity score matching

Propensity score matching yielded 245 pairs with balanced baseline and operative covariates, except for iatrogenic dissection and concomitant mitral/tricuspid valve surgery, which were uncommon in this series (*Table 1*).

**Fig. 2:**
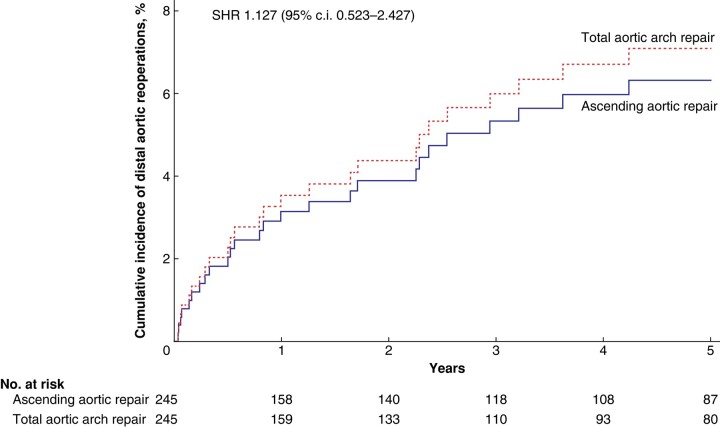
Cumulative incidences of distal aortic reoperations after ascending aortic repair and total aortic arch repair for acute DeBakey type 1 aortic dissection.

Among 245 propensity score-matched pairs, total aortic arch repair did not decrease the incidence of distal aortic reoperation (at 5 years, cumulative incidence 6.7% *versus* 6.7%, SHR 1.127, 95% c.i. 0.523 to 2.427; *Fig. 2*). Such a difference did not change at the 10-year interval (cumulative incidence 11.4% *versus* 9.1%, SHR 1.132, 95% c.i. 0.582 to 2.202). Total aortic arch repair was associated with an increased incidence of postoperative stroke/global brain ischaemia (25.7% *versus* 18.4%, *P* = 0.050) and dialysis (19.6% *versus* 12.7%, *P* = 0.003). The numerically higher rate of in-hospital mortality (16.3% *versus* 11.8%, *P* = 0.153) contributed to the increased incidence of composite outcome (33.9% *versus* 25.3%, *P* = 0.038) after total aortic arch repair compared to ascending aortic repair. At 5 years, mortality and cumulative incidence of proximal aortic reoperations were comparable in the study groups (*Table 3*).

### Ascending aortic repair *versus* conventional total aortic arch repair *versus* frozen elephant trunk repair multivariable adjusted analysis

Analysis of different aortic arch repair techniques was performed by multivariable adjusted analysis because of the relatively limited number of patients treated with conventional total aortic arch repair or the frozen elephant trunk repair (*Table 4*). Patients of these three study groups had a comparable prevalence of co-morbidities, but increased prevalence of spinal malperfusion in the frozen elephant trunk repair group and of tear in the aortic arch in both total aortic arch repair groups.

**Table 4:** Patients’ characteristics and operative data of patients who underwent ascending aortic repair, conventional total aortic arch repair or total aortic arch repair with the frozen elephant trunk technique

Variables	Ascending aortic repair	Conventional total aortic arch repair	Frozen elephant trunk repair	*P*
	(*n* = 915)	(*n* = 156)	(*n* = 128)	
**Baseline characteristics**				
Age (years), mean(s.d.)	50.5(7.6)	49.3(8.6)	49.6(8.2)	0.236
Female	176 (19.2)	21 (13.5)	22 (17.2)	0.214
eGFR, mean(s.d.), ml/min 1.73 m^2^	75(24)	78(25)	80(26)	0.080
Genetic aortic syndrome	39 (4.3)	9 (5.8)	7 (5.5)	0.623
Bicuspid aortic valve	64 (7.0)	9 (5.8)	4 (3.1)	0.232
Iatrogenic dissection	14 (1.5)	0 (0)	0 (0)	0.111
Diabetes	17 (1.9)	5 (3.2)	2 (1.6)	0.503
Stroke	31 (3.4)	1 (0.6)	5 (3.9)	0.158
Pulmonary disease	49 (5.4)	5 (3.2)	4 (3.1)	0.324
Extracardiac arteriopathy	27 (3.0)	3 (1.9)	5 (3.9)	0.610
Prior cardiac surgery	12 (1.3)	3 (1.9)	2 (1.6)	0.828
Cardiac massage	35 (3.8)	5 (3.2)	5 (3.9)	0.927
Shock requiring inotropes	123 (13.4)	23 (14.7)	20 (15.6)	0.752
Invasive mechanical ventilation	73 (8.0)	10 (6.4)	10 (7.8)	0.795
Preoperative malperfusion				
Cerebral	197 (21.5)	39 (25.0)	28 (21.9)	0.626
Spinal	23 (2.5)	3 (1.9)	10 (7.8)	0.003
Renal	87 (9.5)	15 (9.6)	18 (14.1)	0.270
Mesenteric	40 (4.4)	9 (5.8)	8 (6.3)	0.526
Peripheral	175 (19.1)	30 (19.2)	20 (15.6)	0.629
**Operative data**				
Salvage procedure	36 (3.9)	5 (3.2)	5 (3.9)	0.908
Tear in the aortic arch	101 (11.0)	73 (46.8)	42 (32.8)	<0.001
Aortic root replacement	357 (39.0)	46 (29.5)	46 (35.9)	0.070
Coronary surgery	89 (9.7)	8 (5.1)	10 (7.8)	0.158
Mitral or tricuspid valve surgery	3 (0.3)	0 (0)	0 (0)	0.627
Aortic cross-clamp time, mean(s.d.), min	119(56)	164(69)	164(79)	<0.001
Cardiopulmonary bypass time, mean(s.d.), min	213(83)	279(92)	263(110)	<0.001

Values are *n* (%) unless otherwise indicated. eGFR: estimated glomerular filtration rate according to the CKD-EPI equation.

Cumulative incidence of distal aortic reoperation did not significantly differ between the study groups but was numerically lower after the frozen elephant trunk repair (*Table 5*).

**Table 5:** Early and late outcomes with adjusted risk estimates of patients who underwent ascending aortic repair, conventional total aortic arch repair or total aortic arch repair with the frozen elephant trunk technique

	Ascending aortic repair	Conventional total aortic arch repair	Frozen elephant trunk repair
	(*n* = 915)	(*n* = 156)	(*n* = 128)
**Early outcomes**			
In-hospital death	109 (11.9)	21 (13.5)	21 (16.4)
		1.204, 0.677–2.142	1.404, 0.783–2.515
Stroke/global brain ischaemia	177 (19.3)	29 (18.6)	39 (30.5)
		1.045, 0.618–1.768	2.869, 1.770–4.650
Composite outcome	238 (26.0)	43 (27.6)	46 (35.9)
		1.277, 0.805–2.024	2.269, 1.438–3.580
Paraparesis/paraplegia	51 (5.6)	13 (8.3)	10 (7.8)
		1.556, 0.792–3.056	1.543, 0.732–3.249
Mesenteric ischaemia	38 (4.2)	6 (3.8)	5 (3.9)
		1.196, 0.426–3.358	1.087, 0.342–3.448
Sepsis	112 (12.2)	29 (18.6)	13 (10.2)
		1.865, 1.108–3.138	0.766, 0.398–1.473
Dialysis	119 (13.0)	32 (20.5)	25 (19.5)
		1.423, 0.819–2.473	1.948, 1.079–3.520
Reoperation for bleeding	118 (12.9)	25 (16.0)	17 (13.3)
		1.392, 0.825–2.348	0.997, 0.546–1.822
Heart failure	110 (12.0)	22 (14.1)	14 (10.9)
		1.343, 0.786–2.296	0.978, 0.514–1.859
**5-year outcomes**			
Distal aortic reoperation	49 (6.8)	13 (9.1)	3 (2.4)
		1.613, 0.842–3.089	0.506, 0.149–1.714
Proximal aortic reoperation	34 (4.8)	4 (2.7)	2 (1.6)
		0.570, 0.203–1.602	0.453, 0.108–1.898
Mortality	168 (21.2)	36 (23.9)	29 (31.1)
		1.215, 0.821–1.800	1.783, 1.168–2.721

Values are counts, rates or cumulative incidences with percentages (in parentheses). Composite outcome: in-hospital death, stroke and/or global brain ischaemia. Risk estimates are odds ratios, subdistributional hazard ratio and hazard ratios with 95% confidence intervals as adjusted by multiple baseline and operative covariates.

In multivariable adjusted analysis, patients who underwent the frozen elephant trunk repair had a significantly higher incidence of stroke/global brain ischaemia (adjusted OR 2.869, 95% c.i. 1.770 to 4.650), composite outcome (adjusted OR 2.269, 95% c.i. 1.438 to 3.580) and dialysis (adjusted OR 1.948, 95% c.i. 1.079 to 3.520) compared to patients who underwent ascending aortic repair. The increased rate of neurological complications after the frozen elephant trunk repair was mainly due to a higher incidence of suture of the left subclavian artery without its revascularization in this group compared to the conventional total aortic arch repair (10.2% *versus* 6.4%), which led to a significantly higher incidence of postoperative stroke/global brain ischaemia (53.8% *versus* 0%, *P* = 0.007). Furthermore, patients who underwent the frozen elephant trunk procedure had a higher incidence of haemorrhagic stroke compared to ascending aortic repair and conventional total aortic arch repair (6.3% *versus* 1.5% *versus* 0.6% respectively, *P* = 0.003). The frozen elephant trunk repair was also associated with increased late mortality (adjusted HR 1.783, 95% c.i. 1.168 to 2.721; *Table 5*). Patients who underwent conventional total aortic arch repair had similar early and late outcomes compared to those who underwent ascending aortic repair, but an increased rate of postoperative sepsis (adjusted OR 1.865, 95% c.i. 1.108 to 3.138; *Table 5*). Early adverse events were numerically less frequent after ascending aortic repair than in both total aortic arch repair techniques, but these differences did not reach statistical significance.

## Discussion

In this study of patients under 60 years old, total surgical repair of the aortic arch for DeBakey type 1 aortic dissection did not reduce the incidence of distal aortic operations at 5 years. Total aortic arch repair was associated with an increased incidence of neurological complications and renal failure, and frozen elephant trunk repair of the aortic arch was associated with an increased risk of mid-term mortality.

DeBakey type 1 aortic dissection is an emergency condition that poses the dilemma of whether to accomplish an expedited surgical repair limited to the ascending aorta with or without a bevelled distal anastomosis, that is the hemiarch repair, or to repair the entire aortic arch addressing or not the dissected descending thoracic aorta. These surgical approaches differ significantly in terms of technical complexity, need for strategies for prolonged cerebral protection and, to some extent, risk of early postoperative adverse events. Indeed, four pooled analyses confirmed that ascending aortic repair is associated with a significantly lower risk of early mortality compared to extensive aortic arch repair^1,9–11^. On the contrary, these studies reported equivocal results regarding the incidence of late aortic reoperations and mortality after proximal compared to extended aortic surgery^1,9–11^. However, these pooled survival analyses suffer from methodological limitations related to the lack of results adjusted for confounders and of cumulative incidences of aortic reoperations estimated by competing risk analysis and methods not addressing the time-to-event nature of survival analyses. Importantly, pooled analyses included patients from Asia, whose early and late results are excellent and are not replicated in Western countries^12,13^.

The ERTAAD study showed that patients under 60 years old with DeBakey type 1 aortic dissection had a significantly higher incidence of distal aortic reoperation compared to older patients. It is hypothesized that this might be partly due to higher late mortality of these patients as well as to a more conservative strategy in the elderly with aortic wall degeneration due to their increased operative risk. Furthermore, younger patients may have more aggressive conditions leading to aortic dissection compared to older patients. Patients who underwent ascending aortic repair had a rather low operative mortality and the rates of adverse events were numerically lower than in patients who underwent total aortic arch repair. It is worth noting that, despite the technique employed for aortic repair, the cumulative incidence of distal aortic reoperation at 5 years was below 10% in all study groups, with a numerically lower incidence of distal aortic operation after frozen elephant trunk aortic repair (2.4%). Controversial results on the durability of aortic repair have been reported in a few studies evaluating young patients with TAAD. Among 51 TAAD patients younger than 50 years old, Uehara *et al.*^3^ reported distal aortic reoperations in 25% of patients after total aortic arch repair and in 17% of patients after hemiarch repair. However, 27.5% of their patients had a connective tissue disorder. Tamura *et al.*^4^ reported the results of 31 TAAD patients under 50 years old. Total aortic arch replacement was performed in 68% of patients and at 5 years distal aortic reoperation had been required in 8% of them. Piccardo *et al.*^6^ reported the results of 50 TAAD patients aged under 50 years old. After a mean follow-up of 5.1 years, 12% of patients required a distal aortic reoperation, but early postoperative mortality was 24%. Ma *et al.*^5^ performed a frozen elephant trunk repair in 518 TAAD patients with a mean(s.d.) age of 46.2(10.5) years. Twelve-year mortality was 28.0% and cumulative incidence of distal reoperations was 8.5%.

With the uncertainties around the efficacy of total aortic arch repair to prevent distal aortic complications^14^, it is not a surprise that in the recent large UK National Adult Cardiac Surgical Audit database on TAAD, only 3% of patients underwent aortic arch replacement^15^. Still, current guidelines support a policy of extensive repair for DeBakey type 1 aortic dissection. In 2022, the American College of Cardiology/American Heart Association guideline supported a policy of extended aortic repair with antegrade stenting of the proximal descending thoracic aorta in patients with acute type A aortic dissection and dissection flap extending through the arch into the descending thoracic aorta^16^. Similarly, in 2019, the European Association for Cardio-Thoracic Surgery (EACTS) and the European Society for Vascular Surgery expert consensus suggested that the frozen elephant trunk repair may be considered for use in patients undergoing surgery for acute TAAD^17^. It is recognized that extended aortic arch repair is indicated when the tear involves the distal part of the aortic arch, in the presence of aneurysm and in those patients with significant injury of the aortic arch wall. However, there are not sufficient data to support a policy of extensive aortic arch repair in the absence of significant injury. Indeed, the present data are concordant with those of the NORCAAD registry, which confirmed the long-term durability of ascending aortic repair independently of the extent of dissection^18^. In the setting of a multicentre study with heterogeneous interinstitutional and between-surgeons’ strategies of surgical treatment, it is not feasible to explore the individual choice that led to a conservative or extended surgical aortic repair in the present series. However, in this series, only 40.5% of patients who underwent total aortic arch replacement for DeBakey type 1 aortic dissection had a tear of the aortic arch. It is hypothesized that a strategy of total aortic arch replacement was most often dictated only by the presence of dissection involving the aortic arch.

The present results should be viewed considering several limitations which deserve to be acknowledged. First, the retrospective nature of the ERTAAD is the major limitation of the study. Second, the case mix was addressed using multilevel mixed-effects multivariable regression methods, but this might not completely prevent the bias associated with the clustering effect of participating hospitals. Third, analysis of late outcomes was limited to 5 years, but this study interval may not be sufficient to evaluate the natural history of dissected/diseased distal aorta in patients with a long life expectancy. Fourth, the incidence of distal aortic reoperation does not completely reflect the incidence of complications related to aortic dissection such as aortic-related death or any aortic complication left untreated. Fifth, the present series is not powered to detect significant differences for the reported clinical outcomes. In fact, with an in-hospital mortality difference of 11.8% and 16.3% as observed in the propensity score-matched groups, 940 patients would have been needed per group to reject the null hypothesis. Sixth, in this series two patients underwent endovascular reintervention on the axillary artery and on the renal artery. Both of these patients were from the ascending aortic repair group. It is believed that these procedures should be considered within the spectrum of reoperations after surgery of TAAD. Indeed, if the aim of extensive aortic repair is aortic remodelling, failure to achieve/maintain patency of the main aortic branches is an adverse endpoint of the procedure. The same applies to endovascular embolization of aortic pseudoaneurysms. However, excluding these three reoperations would have resulted in a lower incidence of distal aortic reoperations in the ascending aortic repair group. Seventh, this registry includes consecutive series of patients, but 11 hospitals provided data on their consecutively operated patients after 2005 because of the lack of availability of patients’ records in the early years of the study period. This prevented an interinstitutional analysis of the rates of total aortic arch replacement. Multilevel mixed-effects regression analyses were used to prevent any bias related to any difference in terms of rate of total aortic arch replacement between institutions. Finally, data are not available for those patients in whom aortic arch repair was indicated by extensive tear and/or severe wall degeneration of the aortic arch/descending thoracic aorta. This prevented a more in-depth analysis of the safety and efficacy of aortic arch repair in patients without severely injured aorta.

Further large studies with longer follow-up are needed to evaluate the safety of total aortic arch repair and its efficacy in preventing distal aortic degeneration in young patients with acute DeBakey type 1 aortic dissection.

## Data Availability

Data of this registry are not publicly available due to privacy restrictions.
